# A MEMS traveling-wave micromotor-based miniature gyrocompass

**DOI:** 10.1038/s41378-025-00868-9

**Published:** 2025-02-18

**Authors:** Jinxuan Xie, Tong Zhou, Yu Chen, Yi Zhou, Bo Jiang, Jing Zhang, Zhenjun Wang, Wentao Li, Han Sun, Xuyang Zhu, Xiaoshi Li, Tianyu Yang, Yan Su

**Affiliations:** 1 School of Mechanical Engineering, Nanjing University of Science and Technology, Nanjing, China; 2 Jiangsu Research Center for Intelligent Navigation and Control of Complex Motion, Nanjing, China; 3 Microsystem and Terahertz Research Center, China Academy of Engineering Physics, Chengdu, China; 4 Institute of Electronic Engineering, China Academy of Engineering Physics, Mianyang, China

**Keywords:** Electrical and electronic engineering, Sensors

## Abstract

Traditional gyrocompasses, while capable of providing autonomous directional guidance and path correction, face limitations in widespread applications due to their large size, making them unsuitable for compact devices. Microelectromechanical system (MEMS) gyrocompasses offer a promising alternative for miniaturization. However, current MEMS gyrocompasses require the integration of motor rotation modulation technology to achieve high-precision north-finding, whereas conventional motors in previous research introduce large volume and residual magnetism, thus undermining their size advantage. Here, we innovatively propose a miniature MEMS gyrocompass based on a MEMS traveling-wave micromotor, featuring the first integration of a chip-scale rotational actuator and combined with a precise multi-position braking control system, enabling high accuracy and fast north-finding. The proposed gyrocompass made significant advancements, reducing its size to 50 × 42.5 × 24.5 mm³ and achieving an azimuth accuracy of 0.199° within 2 min, which is half the volume of the smallest existing similar devices while offering twice the performance. These improvements indicate that the proposed gyrocompass is suitable for applications in indoor industrial robotics, autonomous driving, and other related fields requiring precise directional guidance.

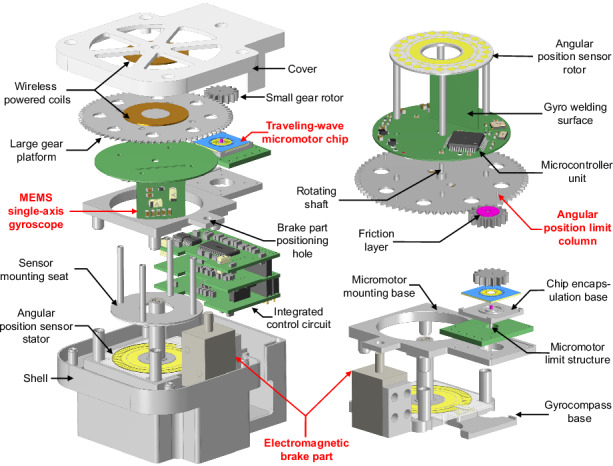

## Introduction

Since antiquity, the determination of direction has always been an essential part of human life. The azimuth is the clockwise angle between an object’s direction and true north, its measurement provides crucial reference information for positioning and navigation systems^1,2^. With the advancement of technology, the determination of the azimuth has evolved into a technical challenge requiring high precision and reliability^3^. This is especially true in fields such as geological exploration^4^, vehicle orientation^5,6^, marine industries^7^, and inertial guidance^8^. The gyrocompass, also known as the gyro north-finder, is one of the significant achievements in the application of inertial technology. Unlike north-finding systems based on Earth’s magnetic north^9,10^, celestial observation north-finding systems^11,12^, and global navigation satellite system–based north-finding systems^13–15^, a gyrocompass determines the azimuth measurement by measuring the Earth’s rotational angular velocity using a gyroscope (gyro). This method has the advantages of being omnidirectional, all-weather, and highly autonomous^16^. A gyrocompass can operate independently and is less susceptible to external information interference, which provides a reliable solution for orientation in complex and harsh environments^17,18^. Since the Earth’s rotational angular velocity is only approximately 15°/h, a traditional gyrocompass generally uses high-precision dynamically tuned gyroscopes, laser gyroscopes, fiber optic gyroscopes, and hemispherical resonator gyroscopes to ensure high north-finding accuracy^19–23^. Autonomous orientation and navigation without satellite assistance is a critical challenge that needs to be addressed, as it limits the widespread use of indoor industrial robotics, autonomous driving, and similar fields. However, the large size and high cost of these gyroscopes limit the embedded applications of gyrocompasses in the aforementioned fields^24,25^. Smaller, higher-performance gyrocompasses could bring revolutionary changes to satellite-independent directional navigation.

In contrast, MEMS gyros offer attractive advantages in terms of size, weight, power consumption, and cost, enabling the miniaturization and handheld design of gyrocompasses^26,27^. Nonetheless, the long-term bias drift and relatively lower performance of MEMS gyros make it difficult to achieve high-precision azimuth measurements relying solely on these gyros^28,29^. To ensure the north-finding accuracy and long-term effectiveness of a MEMS gyrocompass, the Carouselin/Maytagging methods have garnered attention as system-level rotational modulation self-compensation techniques^30–35^. The Carouseling method involves mounting a gyro on a rotating platform with a constant speed, allowing for the determination and elimination of gyroscopic bias error, scale factor error, and drift error through computation and thereby enhancing the accuracy and reliability of azimuth acquisition^27,33^. Such as a Carouseling north-finding system employs a silicon MEMS quadruple mass gyro fixed on a rate table rotating at a constant speed^24^. Although the gyro’s bias instability (BI) is only 0.11°/h, the Carouseling method demonstrates robustness against bias and scale factors variations, achieving a north-finding accuracy of 0.229° with 6 min. However, the Carouseling method requires a complex rotation system with high detection and control precision, which is unsuitable for miniaturized north-finding systems. The Maytagging method, which is often referred to as the static north-finding method, eliminates biases by differentially processing the gyroscopic output from multiple positions^24^. For example, a MEMS gyrocompass with dimensions of 76 × 62 × 26 mm³ utilizes a two-phase axial flux permanent magnet synchronous motor (PMSM) packaged within a printed circuit board to drive the inertial measurement unit (IMU) rotation^25^. By employing a four-position Maytagging method, the above gyrocompass can achieve a north-finding accuracy of 0.5° within 3 min with a gyro BI of 0.027°/h.

Another method, the virtual Carouseling/Maytagging mitigates the inherent bias and drift in gyro by adjusting operational modes or rotating the sensitive axis, thereby enhancing performance and long-term stability^29,36–38^. A virtual Carouseling method for a whole-angle (WA) hemispherical resonator gyroscope (HRG) applies a virtual Coriolis voltage to the sense mode and realizes standing wave self-precession relative to the resonator^38^. Using this virtual Carouseling method, the WA HRG achieves self-calibration and self-compensation of various errors, and the BI is reduced by one order, from 0.0991°/h to 0.0092°/h. These enhancements enable the WA HRG to quickly and accurately measure the Earth rotation component^38^. The two-position virtual Maytagging (VM) for MEMS gyrocompass, uses an electronically controlled vibrating honeycomb disk resonator gyroscope mode, eliminating the need for mechanical rotating parts^29^. This gyro achieves a BI of 0.0078°/h, and the gyrocompass prototype, including the control circuit, measures 60 × 60 × 60 mm³. Under VM, it achieves a north-finding accuracy of 0.204° within 5 min, whereas the traditional Maytagging method based on a rate turntable achieves a north-finding accuracy of 0.172°. The virtual Carouseling/Maytagging method allows for the self-calibration and self-compensation of gyro errors without needing a turntable or external references, which can radically enhance the gyrocompass’s performance. Whereas, the virtual Carouseling/Maytagging method requires more precise control of the gyro’s operation modes and real-time feedback modulation, which may necessitate a more sophisticated system and increased computational resources. The Maytagging method does not require processing the gyro itself, providing a relatively straightforward and easy-to-implement method to enhance the performance of MEMS gyrocompasses. However, the commonly used rotational modulation actuators in current gyrocompasses are composed of traditional electromagnetic mechanisms, which are much larger than MEMS gyro chips. This size disparity limits the further integration of high-precision MEMS gyrocompasses and prevents them from fully showcasing the high integration and chip-scale advantages of MEMS technology^25,29^. Therefore, the miniaturization of the rotational actuator motor remains a bottleneck in the development of a gyrocompass.

A MEMS traveling-wave micromotor (MTWMM) is a millimeter-scale micro-actuator chip manufactured using MEMS technology, belonging to the micro-nano domain. An MTWMM generates a driving force to the rotor through the inverse piezoelectric effect of lead zirconate titanate (PZT) and friction, without introducing additional electromagnetic noise interference, and is also less susceptible to interference from non-resonant frequency electric and magnetic fields as a non-magnetic resonant device. This makes an MTWMM well-suited to meet the miniaturization needs of various devices requiring rotational mechanisms^39–42^. After years of research, MTWMMs with an outer diameter of less than 5 mm and a thickness of less than 0.5 mm can output a driving torque of up to 39.4 μN ∙ m^43^, making them ideal candidates for the miniaturization of MEMS gyrocompasses. However, the friction-driven method exhibits significant non-linearity and uncontrollability, making the precise angular control of an MTWMM extremely challenging^44–46^. This difficulty hinders the effectiveness of rotational modulation compensation. Although we have researched capacitive angular position sensors for MTWMMs^47,48^, there are still bottlenecks in achieving precise angular control. Consequently, MTWMMs remain at the experimental research stage, and various challenges have prevented them from being practically applied.

Here, we introduce a highly integrated, miniature, high-performance MEMS gyrocompass that utilizes a MEMS traveling-wave micromotor as the rotational actuator and combines this with a four-position Maytagging method. We utilize a single-axis MEMS gyro chip, along with wireless power and non-contact data transmission technologies, to create a high-density, low-friction-resistance rotational modulation platform. The MTWMM drives a small gear rotor that engages with the rotational modulation platform, achieving the efficient rotation of the MEMS gyro in a highly integrated system. Additionally, by leveraging the friction-driven characteristics of the MTWMM, we innovatively develop a multi-position braking control system to ensure the precise control of the MTWMM and effective compensation with the Maytagging method. To our knowledge, our proposed gyrocompass is currently the smallest MEMS gyrocompass based on rotational modulation. This gyrocompass achieves high north-finding accuracy within a short period and outperforms similar devices with integrated motors.

## Results

### Structure design of the gyrocompass

**Fig. 1:**
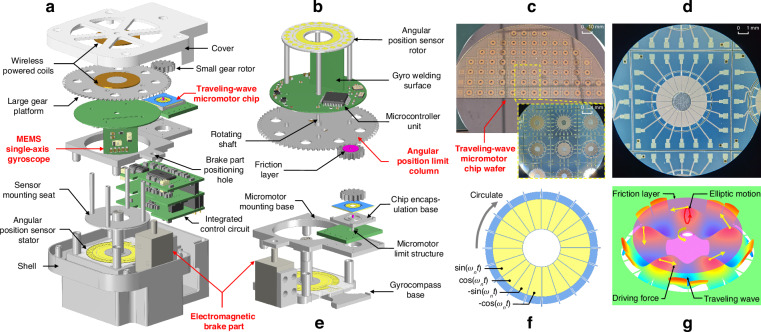
Structural model of the proposed gyrocompass and the microelectromechanical system traveling-wave micromotor (MTWMM). **a** Exploded view of the overall structure. **b** Structural model of the rotational modulation platform (including the inertial measurement unit). **c** An MTWMM chip wafer. **d** An individual MTWMM chip located on the wafer under a microscope. **e** Model of the internal base, limiting structure, and brake part of the proposed gyrocompass. **f** Sequence of four orthogonal drive signals for the MTWMM micro-stator. **g** Elliptical motion of the surface particles on the MTWMM micro-stator and the friction-driven principle

The overall structure of the proposed gyrocompass based on the MEMS traveling-wave micromotor can be referenced in Fig. 1a. The gyrocompass consists of an MTWMM, a rotational modulation platform, a MEMS capacitive angular position sensor, a segmented motor chip mounting base, an electromagnetic brake part, a wireless powered unit, and an integrated control circuit, all enclosed by a shell and cover. The detection of the azimuth angle can be accomplished simply by powering the integrated control circuit and inputting the control commands utilizing the software of the upper computer. As shown in Fig. 1b, to ensure that the gyro-sensitive axis is parallel to the ground, a MEMS single-axis gyro is vertically mounted on a circular base circuit, forming the IMU. This unit is positioned on a large gear platform and secured in place on the platform by a sensor mounting seat with an angular position sensor rotor, collectively forming the rotational modulation platform (RMP).

The MTWMM is machining using MEMS technology, with the resulting chip wafer shown in Fig. 1c (for the machining processes, please refer to the Supplementary Information). After wafer dicing, any individual chip can be extracted for packaging and testing, as shown in Fig. 1d. As shown in Fig. 1e, the MTWMM is packaged on a chip encapsulation base and connected to the external circuitry with gold wire to achieve the four sinusoidal drive signal inputs illustrated in Fig. 1f. The chip encapsulation base is also positioned on the micromotor mounting base, which is positioned on the gyrocompass base. This ensures the precise engagement between the small gear rotor and the RMP, which achieves a reducer-like torque amplification. All of the metal mechanical components are manufactured with a precision of 5 μm. The positioning of each component ensures concentricity and parallelism. The RMP is innovatively mounted in an inverted manner on the gyrocompass base with the assembly of the rotating shaft and bearing, further increasing the integration and reducing the height of the gyrocompass. The integrated control circuit leads a ground wire connected to the shell, and the metal mechanical component of the gyrocompass (such as the shell, large gear platform, and various bases) are tightly connected through metal screws or shaft-hole fittings, forming a conductive path. This provides a grounded shielding structure for the RMP, reducing the impact of external electric field interference.

### The driving principle of the MEMS traveling-wave micromotor

As shown in Fig. 1f, by controlling the driving potentials between each set of four electrodes to maintain a 90° phase difference, two orthogonal standing waves are sequentially formed on the motor micro-stator, as described by Eq. (1). The superposition of these standing waves generates a traveling wave, as described by Eq. (2)^41^:

1 $$\left\{\begin{array}{l}{u}_{1}={A}_{z}\,\sin (k\theta)\sin ({\omega}_{n}t)\\ {u}_{2}={A}_{z}\,\sin (k\left(\right.\theta +\lambda /4)\sin ({\omega }_{n}t\pm \pi /2)\end{array}\right.$$

2 $$u={u}_{1}+{u}_{2}={A}_{z}\,\cos (k\theta \mp {\omega }_{n}t)$$

Where *A*_*z*_ represents the amplitude of the standing wave, which is theoretically proportional to the driving voltage amplitude. *θ* is the angular displacement; *λ* is the angular displacement of the standing wave wavelength, with *λ* = 2*π*/*n*; *k* = 2*π*/*λ*; and *ω*_*n*_ is the resonant frequency of the standing wave mode.

Due to the traveling wave motion, there are always *n* pairs of wave peaks and troughs present at any given time. The peaks rotate circumferentially along the micro-stator, which causes an elliptical motion opposite to the direction of wave propagation^40–43^, as shown in Fig. 1g and Supplementary Fig. 2. The surface particles of the traveling wave and the friction layer experience a velocity difference. This generates a symmetric driving force around the axis through sliding friction and thereby drives the friction layer’s rotation. As shown in Fig. 1b, the friction layer is assembled with a small gear rotor. Therefore, by applying four sinusoidal drive signals with a 90° phase difference, the small gear rotor can be driven to rotate.

In our study, we explore the practical application of an MTWMM for the first time using a micromotor chip with a micro-stator’s outer diameter of 6 mm and a thickness of less than 0.5 mm. The driving state of the MTWMM can be referenced in Supplementary Movie 1.

### The north-finding principle of the gyrocompass

**Fig. 2:**
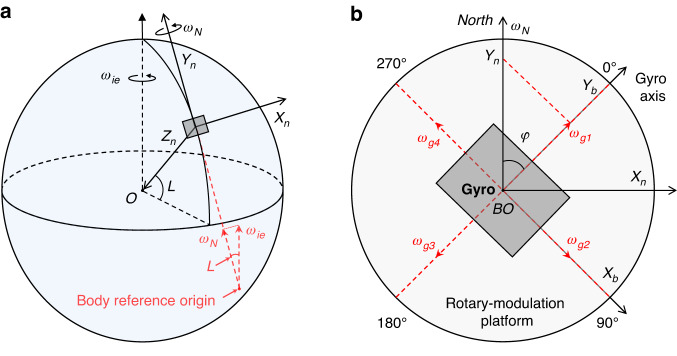
The north-finding principle. **a** Earth model and navigation reference frame (North-East-Down reference frame, i.e., *<n>* frame). **b** Four-position Maytagging north-finding principle and body reference frame

As shown in Fig. 2a, the navigation reference frame (*<n>* frame) has its origin at the intersection point *O* of the line connecting the center of the Earth and the body. The local horizontal plane is used as the reference plane. In the *<n>* frame, the *X*_*n*_ axis points east from the body reference origin (*BO*), the *Y*_*n*_ axis points north, and the *Z*_*n*_ axis points down, forming a North-East-Down reference frame^5^. The true north that the north-finding system seeks is the *Y*_*n*_ axis. The azimuth angle is the angle *φ* between the gyro-sensitive axis (*Y*_*b*_) in the body reference frame (*<b>* frame) shown in Fig. 2b and the *Yn* axis. As depicted in Fig. 2a, at a local latitude *L*, the component of the Earth’s rotational angular velocity (*ω*_*ie*_) in the *<n>* frame is

3 $${\omega }_{ie}^{n}=[\begin{array}{ccc}{\omega }_{E} & {\omega }_{N} & {\omega }_{D}\end{array}]=[\begin{array}{ccc}0 & {\omega }_{ie}\,\cos L & {\omega }_{ie}\,\sin L\end{array}]$$

As shown in Fig. 2b, in the four-position Maytagging north-finding system, the gyro is sensitive to the Earth’s rotational angular velocity components at four positions, each differing by 90° in the angular position:

4 $$\left\{\begin{array}{ll}{\omega }_{g1}={\omega }_{0}+K{\omega }_{N}\,\cos \varphi +\varepsilon ({\tau }_{1})\\ {\omega }_{g2}={\omega }_{0}-K{\omega }_{N}\,\sin \varphi +\varepsilon ({\tau }_{2})\\ {\omega }_{g3}={\omega }_{0}-K{\omega }_{N}\,\cos \varphi +\varepsilon ({\tau }_{3})\\ {\omega }_{g4}={\omega }_{0}+K{\omega }_{N}\,\sin \varphi +\varepsilon ({\tau }_{4})\end{array}\right.$$

Where *ω*_0_ represents the bias of the MEMS gyro. Althoughthis parameter exhibits some instability^29^, the bias at the four positions during the short-term north-finding process can be considered consistent. *K* is the scale factor of the MEMS gyro, and *ε*(*τ*) is the random drift in the gyro output, which can be neglected in subsequent calculations. The impact of this drift on the north-finding accuracy can be referenced in Eq. (8). By subtracting two outputs of the gyro separated by 180°, the following expressions are obtained:

5 $$\left\{\begin{array}{ll}{\omega }_{g1}-{\omega }_{g3}=2K{\omega }_{ie}\,\cos L\,\cos \varphi +\varepsilon ({\tau }_{1})-\varepsilon ({\tau }_{3})\\ {\omega }_{g4}-{\omega }_{g2}=2K{\omega }_{ie}\,\cos L\,\sin \varphi +\varepsilon ({\tau }_{4})-\varepsilon ({\tau }_{2})\end{array}\right.$$

Neglecting the random drift, dividing the two equations yields

6 $$\frac{{\omega }_{g4}-{\omega }_{g2}}{{\omega }_{g1}-{\omega }_{g3}}=\frac{2K{\omega }_{ie}\,\cos L\,\sin \varphi }{2K{\omega }_{ie}\,\cos L\,\cos \varphi }$$

Therefore, the azimuth angle is

7 $$\varphi =\arctan \frac{{\omega }_{g4}-{\omega }_{g2}}{{\omega }_{g1}-{\omega }_{g3}}$$

As indicated by Eqs. (5–7), the four-position Maytagging method compensates for the MEMS gyro bias instability by differentiating the outputs of the gyros at symmetric positions, and the effects of the latitude and scale factor on the north-finding accuracy can largely be ignored. This method is suitable for environments for which the geographic location is unknown or difficult to obtain because it provides a simple, efficient, and accurate way to determine true north.

### Hardware and system of the gyrocompass

**Fig. 3:**
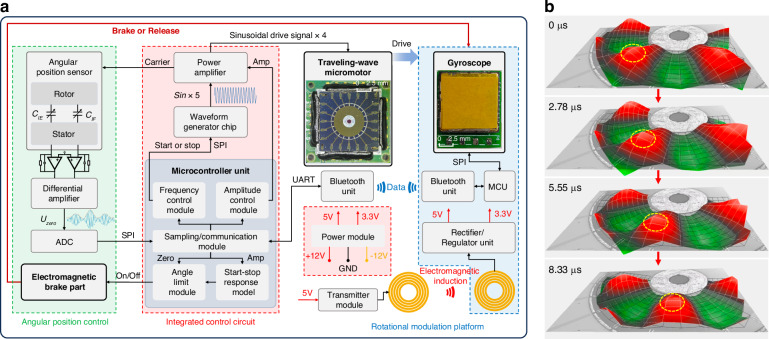
The proposed gyrocompass’s hardware and the traveling wave. **a** The proposed gyrocompass’ hardware and system, which primarily includes the following three components: the integrated control circuit, the angular position control module, and the rotational modulation platform. **b** The traveling wave excited on the micro-stator observed using a microscopic laser vibrometer, and the yellow dashed circle is used to indicate one peak of the observed traveling wave

As shown in Fig. 1a, the wireless-powered transmission coil and receiving coil are positioned on the cover and the large gear platform, respectively. As illustrated in Fig. 3a, the transmitter module sends a high-frequency electrical signal to the transmission coil. Based on the principle of electromagnetic induction, this electrical signal is transmitted to the receiving coil. The rectifier/regulator unit on the circular base circuit converts the high-frequency electrical signal into the DC voltage required by the MEMS gyro, microcontroller unit (MCU), and Bluetooth unit, thus producing a wireless power supply for the RMP.

The MCU on the integrated control circuit manages the sampling and control tasks of the entire system and establishes a communication link with the upper computer. Since the frequency of the sinusoidal drive signal for the MTWMM is relatively high (above 100 kHz), four high-performance multi-bit parallel digital-to-analog converters (DACs) are typically used to output four highly matched quadrature drive signals^38^. However, multi-bit DACs not only occupy a significant amount of space but also require an MCU with numerous pins for control, which is detrimental to system integration. As shown in Fig. 3a, our proposed MTWMM driving system innovatively utilizes a waveform generator chip instead of DACs. The frequency and phase of the sinusoidal signals can be controlled through a serial peripheral interface (SPI). The signals output from the waveform generator chips are then amplified by the power amplifiers, whose amplification (AMP) is controlled by the MCU to produce four frequency-matched, quadrature sinusoidal drive signals. This setup simplifies and efficiently achieves the drive control of the MTWMM, thus reducing system complexity. As shown in Fig. 3b, the current driving system can effectively excite a traveling wave on the MTWMM micro-stator, with a resonance cycle of approximately 8.33 μs and a resonance frequency of 120.1 kHz.

As shown in Fig. 3a, the sampling/communication module of the MCU in the integrated control circuit transmits commands to the Bluetooth unit through a universal asynchronous receiver transmitter, which is then sent to the Bluetooth unit in the RMP. Upon receiving the sampling start command, the MCU in the RMP reads the angular velocity data from the MEMS gyro chip through SPI. After processing and packaging the angular velocity data, the MCU in the RMP transmits the data through the Bluetooth unit to the MCU in the integrated control circuit and then sends the data to the upper computer software for display and processing. Once the angular velocity data from all four positions have been sampled, the azimuth calculation can be automatically completed based on Eq. (7). The sampling process can be referenced in Supplementary Movie 3.

### Multi-position braking control system

According to Fig. 1g, the MTWMM drives the small gear rotor through friction. In contrast to traditional motors, which can easily burn out during braking, the rotor being braked and stopped is a normal operating state for the MTWMM and does not lead to damage or performance degradation^41,43^. Furthermore, the driving force output of the MTWMM is continuous. Even in the braked state, the MTWMM can still provide a driving force to keep the rotor in the braked position. When the driving force is stopped, the frictional force between the surface of the micro-stator and the friction layer can cause the motor to self-lock. In a relatively static state for the overall system, the rotor will not tend to rotate. Additionally, it is found that by controlling the amplitude and duration of the driving signal, the MTWMM can achieve a certain level of step precision^44,45^. These characteristics make the multi-position braking control of the MTWMM possible.

**Fig. 4:**
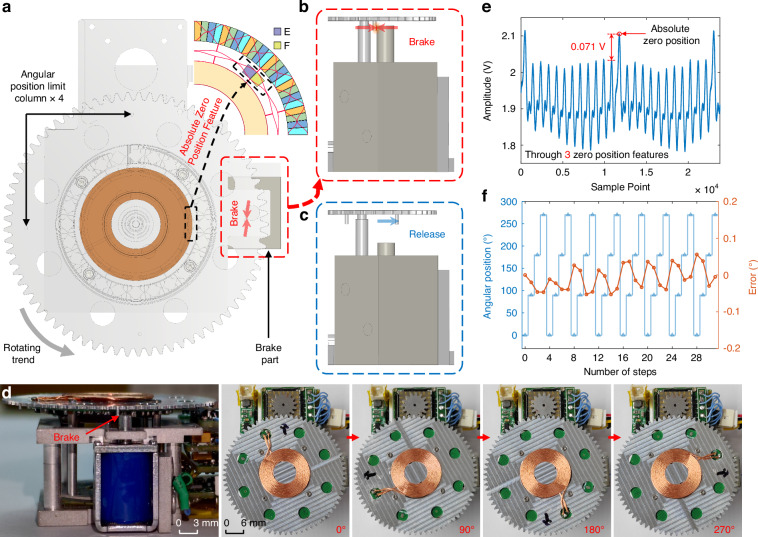
Multi-position braking control model and test results. **a** Overall model of the multi-position braking control system. **b** Angular position limit column is braked by the stop column, which stops the motion of the RMP and suppresses its rotating trend, keeping the RMP at its current position. **c** When the stop column is pulled back and the angular position limit column is released, the MTWMM drives the RMP to rotate. **d** Schematic of the actual braking state and the RMP at the 0°, 90°, 180°, and 270° positions. **e** Absolute zero position detection performance of the capacitive angular sensor (which passes through three absolute zero point features). **f** Precision test result for multi-position braking control after rotation for eight revolutions

As shown in Fig. 1a and e, an electromagnetic brake part is introduced in the gyrocompass. Similarly, a brake part positioning hole is set in place on the micromotor mounting base to restrain the wobble of the stop column, thereby improving the position control accuracy. As shown in Figs. 1b and 4a, four angular position limit columns, which are evenly distributed on the backside of the large gear platform, are precision-machined to meet the angular position control requirements of the four-position Maytagging method.

After analyzing the startup and stop responses of the MTWMM driving the RMP, we establish a start-stop response model within the integrated control circuit (Fig. 3a). By controlling the MTWMM driving frequency and amplitude from the upper computer, the start-stop response model calculates the MTWMM’s drive duration and the point-in-time for the electromagnetic brake part pop out. When the upper computer sends a step control command, the angle limit module in Fig. 3a controls the electromagnetic brake part to pull back, as shown in Fig. 4c, which releases the angular position limit columns. Immediately afterward, the waveform generator chip is controlled to generate driving signals, which causes the RMP to start rotating. During this process, the sampling of the MEMS gyro is paused.

When the calculated point-in-time is reached, the angle limit module controls the electromagnetic brake part to pop out. At this point, the MTWMM’s drive does not stop. As shown in Fig. 4a, b, the angular position limit column is braked by the stop column, which suppresses the platform’s rotating trend. While the RMP may experience a degree of rebound due to the collision, the continuously applied drive force from the MTWMM causes the RMP to return to the braked position. Once the calculated drive duration is reached, the RMP stabilizes at the braked position, as shown in Supplementary Movie 2. The MTWMM drive is then stopped, which allows for the sampling of the Earth’s rotational angular velocity components at the current position. The entire control process can be automated by the upper computer software, which performs azimuth angle detection strictly according to the set sampling time. After collecting the Earth’s rotational angular velocity components at the four positions shown in Fig. 4d, the current azimuth can be calculated to achieve the pointing function.

Due to the absence of a true mechanical locking mechanism in the MTWMM, its starting position may not be fixed in place after experiencing jolts. This can lead to the failure of the braking control based on the start-stop response model. Additionally, purely open-loop control systems exhibit low robustness. This makes it difficult to avoid risks such as control command failures or motor state changes during automatic north-finding, which can in turn lead to position control issues. To address these concerns, as shown in Figs. 1a, b and 4a, we integrate a capacitive angular position sensor with an absolute zero position feature^47^.

As shown in Figs. 3a and 4a, the waveform generator chip and power amplifier provide an AC sinusoidal carrier to the stator of the angular position sensor. This carrier induces amplitude modulation signals on the inner absolute zero position detection electrodes E and F, with the amplitudes positively correlated with the coupling capacitance. These coupling capacitances are proportional to the overlapping area between the stator and rotor. Since the electrode area of the rotor within the absolute zero position range is twice that of other ranges, a significant and abrupt change in amplitude occurs within this range after C/V conversion and differential amplification^47^. The *U*_*zero*_ waveform after analog-to-digital converter sampling and digital envelope detection is shown in Fig. 4e. The figure indicates that this abrupt change is easy to detect and highly repeatable. In the multi-position braking control system, the first position after passing the absolute zero position is set as the 0° for the gyrocompass. Before the beginning of the north-finding process, the RMP is rotated 360° to locate the zero position and begin sampling from 0°. During sampling, the monitoring of whether the zero position is passed when returning to 0° can be utilized to assess whether the position control is functioning correctly and to determine the effectiveness of the current azimuth result.

Under the condition of a level base, the north-finding error in the four-position Maytagging method has a 1:1 relationship with the angular position control error^49,50^. Therefore, the accuracy of multi-position braking control significantly impacts the azimuth accuracy of the proposed gyrocompass. To ensure the smooth operation of the stop column, the outer diameter of the brake part positioning hole is slightly larger than that of the stop column by *R*_*E*_ = 0.02 mm, which is a significant source of angular position control error. With the angular position limit column having an outer diameter of *R*_*O*_ = 31.2 mm, the theoretically maximum angular positioning error is *E*_*Amax*_ = 0.02 × (360°/31.2*π*) = 0.073°. Since the RMP only rotates in one direction, the one-way wobble range of the stop column is slightly smaller than *R*_*E*_. The test results for the angular position control precision are shown in Fig. 4f. The figure shows that the accuracy for each angular position is 0.024° (1*σ*) for multiple measurements. In future research, we will investigate a closed-loop control system based on the three-ring capacitive angular position sensor to further reduce positioning errors and improve rotational modulation compensation^47^.

### Performance characterization of the MEMS gyro in the proposed gyrocompass

**Fig. 5:**
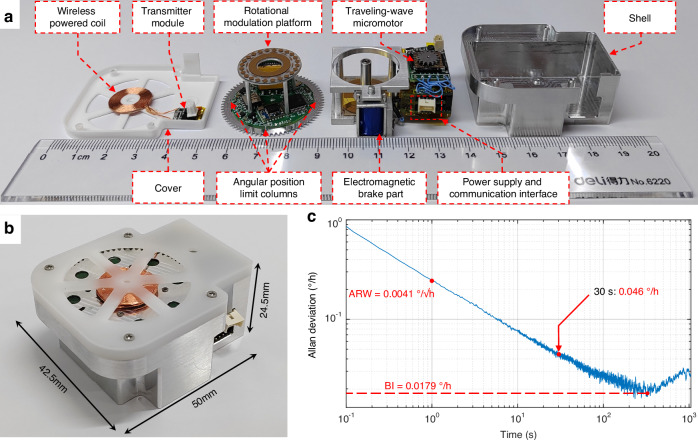
The gyrocompass prototype based on a MEMS traveling-wave micromotor and the performance characterization of the MEMS gyro. **a** Photos of various components in the prototype. **b** Photo of the proposed gyrocompass prototype. The dimensions (including the shell) are 50 × 42.5 × 24.5 mm³. **c** Allan deviation of the MEMS gyro within the overall system at room temperature (with continuous sampling for 200 min)

As illustrated in Fig. 5a, the overall gyrocompass can be divided into four components: the cover, the RMP, the drive control platform, and the shell. After being assembled with the positioning shown in Fig. 1a, the complete MEMS gyrocompass with dimensions of 50 × 42.5 × 24.5 mm³ is obtained, as depicted in Fig. 5b. Notably, the MTWMM does not produce electromagnetic interference and therefore does not affect the MEMS gyro.

To evaluate the performance of the MEMS gyro, we test the gyro within the overall system. The angular velocity data are transmitted from the Bluetooth module to the integrated control circuit and then sent to the upper computer software. The Allan deviation of the MEMS gyro with continuous sampling for 200 min is shown in Fig. 5c. Within the overall system, the MEMS gyro exhibits a BI of 0.0179°/h and an angle random walk of 0.0041°/√h. During multi-position north-finding with a single gyro, the gyro’s random drift cannot be compensated for. Thus, this type of noise affects the north-finding accuracy, as follows^16,34^:

8 $$\sigma (\varphi )=\sqrt{\frac{2}{N}}\frac{\sigma (\tau )}{{\omega }_{N}}({\rm{rad}})$$

where *N* is the number of positions during the north-finding process (e.g., *N* = 4 for the four-position Maytagging method), and *σ*(*τ*) is the Allan deviation corresponding to the MEMS gyro sampling duration *τ*. According to Eq. (8), at a latitude of 31.2° in Nanjing, with the four-position Maytagging method, the deviation of the MEMS gyro of *σ*(*τ*) = 0.046°/h (with *τ* being approximately 30 s) results in an azimuth accuracy of 0.145° (1*σ*) when the gyrocompass is fixed in place.

### Performance test of the proposed gyrocompass

**Fig. 6:**
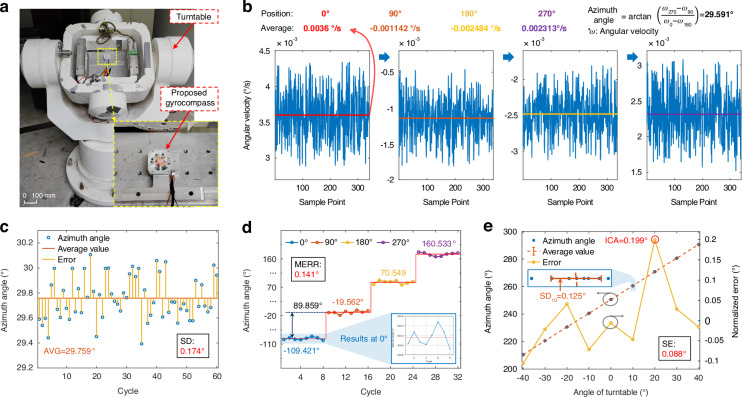
North-finding test results. **a** North-finding test setup. The proposed gyrocompass prototype is fixed in place on a turntable. **b** The angular velocities sensed by the RMP at the four positions of the (0°, 90°, 180°, and 270°) during the first cycle (in **c**) of the north-finding test are shown. Using these data, the azimuth angle can be calculated based on the four-position Maytagging method, resulting in an azimuth angle of 29.591°. **c** Azimuth angle test result at a fixed position. The test parameters are as follows: 2 min (time taken for north-finding) and 60 cycles (number of tests). **d** Azimuth angle test results across the full angular range. For clarity, all azimuth angles greater than 180° have been converted to their corresponding negative angles. The test parameters: 2 min, turntable angles of 0°, 90°, 180°, and 270°, and eight cycles for each position. **e** Internal coincidence accuracy test results. The test parameters: 2 min, turntable angles of 0°, ±10°, ±20°, ±30°, and ±40°, and eight cycles for each position

As shown in Fig. 6a, the north-finding test setup utilizes a three-axis high-precision turntable as the azimuth reference for the gyrocompass. Initially, the proposed gyrocompass based on the MTWMM is mounted on the turntable. After the leveling of the installation plane, no further operations are performed for the turntable. The driving amplitude and frequency of the MTWMM are set utilizing the upper computer software, and the total duration for a single north-finding operation is configured to be 2 min (corresponding to approximately 30 s of sampling time at each position on the RMP). The command to begin azimuth angle detection is then input to the gyrocompass. As shown in Fig. 6b, angular velocity data from the MEMS gyroscope at the four positions of the RMP is sequentially collected and stored, with approximately 340 data points gathered over a 30-s sampling period. After processing and averaging by the upper computer software, the clockwise angle between the 0° position and true north (i.e., the azimuth angle) can be calculated based on the four-position Maytagging method.

As shown in Fig. 6c, a total of 60 tests are conducted, with the average azimuth angle (AVG) being 29.759° and the standard deviation (SD) being 0.174°. The results indicate that the proposed gyrocompass achieves fast and high-precision north-finding at a fixed position. The results also demonstrate that the combination of the MTWMM and the multi-position braking control system ensures high repeatability at each angular position and achieves effective rotational modulation compensation.

Since the orientation of the body constantly changes, testing performed only at fixed positions does not fully represent the performance of our gyrocompass. Therefore, we control the turntable to its absolute 0° and conduct a full angular range test. We detect the azimuth angle at the 0°, 90°, 180°, and 270° positions of the turntable, performing eight tests at each turntable position. The average azimuth angle at each turntable position is used as the reference for comparison. The test results shown in Fig. 6d indicate that when the turntable’s 0° is used as the reference, the maximum error (MERR) relative to the actual rotation angle for the remaining three azimuth angles is 0.141°.

We validate the internal coincidence accuracy (ICA) of the gyrocompass based on the turntable. The turntable is rotated to nine positions (0°, ±10°, ±20°, ±30°, ±40°), with eight samples taken at each position and the north-finding time set to 2 min. The calculation method for the normalized error is as follows. First, the average value of the azimuth angle data for each turntable position is computed. Then, these values are averaged to obtain the normalized mean (the reference of the true value of the azimuth angle at the turntable position of 0°). This normalized mean and the corresponding turntable position are subtracted from each position’s average value to obtain the normalized error for each angle of the turntable. The SD of these normalized errors is used as the standard error (SE) for this test, and the maximum normalized error is taken as the ICA of the proposed gyrocompass. The test results shown in Fig. 6e indicate an ICA of 0.199° and an SE of 0.088°. The standard deviations of the 8-cycle azimuth angle across the nine turntable angles all remain within the range of SD. After approximately six hours of testing, the MTWMM performance did not exhibit any noticeable decline, and the proposed gyrocompass continued to operate normally under the original driving parameters. This demonstrates that the proposed MEMS gyrocompass can perform azimuth angle detection across the full range, providing accurate directional guidance for the body while exhibiting a certain level of stability and durability.

## Discussion

The performance of the reported MEMS gyrocompass is summarized in Table 1. It is evident that the proposed miniature MEMS gyrocompass based on the MEMS traveling-wave micromotor not only achieves high integration for the rotational actuator but also has the smallest dimensions among similar gyrocompasses and a weight of less than 50 g. Additionally, the multi-position braking control system reliably ensures deviation compensation for rotational modulation at four positions. This results in high accuracy and fast north-finding. The MTWMM provides a feasible approach for the further integration of the rotational modulation north-finding system, which makes the proposed lightweight gyrocompass suitable for handheld and embedded applications. Our future work will focus on the development of the Carouseling north-finding method based on the MTWMM and the MEMS three-ring capacitive angular position sensor, with the goal of further improving north-finding performance.

**Table 1:** Performance summaries of the MEMS gyrocompass

Work		34	27	25	35	24	29		This work
Gyro	Number	1	3	1	3	1	1		1
	BI^a^	0.049°/h	0.05°/h	0.027°/h	0.02°/h	0.11°/h	0.0078°/h		0.0179°/h
Size (mm^3^)		100 × 100 × 100	65 × 51 × 35.5	70 × 62 × 26	—	—	60 × 60 × 60		50 × 42.5 × 24.5
Time		1.5 min	3 min	3 min	4 min	6 min	5 min		2 min
Accuracy		1°	0.65°	0.5°	0.229°	0.229°	0.172°	0.204°	0.199°
Method		Maytagging	Carouseling	Maytagging	Maytagging	Carouseling	Maytagging	VM^a^	Maytagging
Rotary actuator		Stepper motor	External turntable	PMSM^a^	External turntable	External turntable	External turntable	—	MEMS traveling-wave micromotor

^a^ *BI* bias instability, *PMSM* permanent magnet synchronous motor, *VM* virtual Maytagging

Furthermore, with the enhancement of the driving force of the MTWMM, we observe that when the IMU shown in Fig. 1b is placed on the small gear rotor, the MTWMM can still drive its rotation without the need for torque amplification through a reducer. This indicates that the integration level of the gyrocompass based on the MTWMM can be further improved. In the future, we will explore high-density integration technology that uses a MEMS gyro as the rotor of an MTWMM, with the goal of developing a chip-sized gyrocompass. It is anticipated that the volume can be reduced to 1/50 of the current size, which could revolutionize the field.

## Conclusion

This paper presents for the first time a MEMS gyrocompass based on a MEMS traveling-wave micromotor. Utilizing an MTWMM as a non-electromagnetic-interference rotating actuator, we develop a complete north-finding system employing a four-position Maytagging method. The system requires only external power and upper computer control for azimuth angle detection, with the prototype reduced to dimensions of 50 × 42.5 × 24.5 mm³. Combining the friction drive characteristics of the MTWMM with the absolute zero position provided by the MEMS capacitive angular position sensor, we establish a multi-position braking control system with a position control accuracy of 0.024°. Within the overall system, the MEMS gyro exhibits a BI of 0.0179°/h. The testing results for the north-finding performance show that with a north-finding time of 2 min, the proposed gyrocompass has an ICA of 0.199° across nine positions and an SD of 0.174° at a fixed position. Our research validates the practicality of the MTWMM and demonstrates that the MTWMM is an ideal actuator for a high-accuracy, miniaturized MEMS gyrocompass, and the MTWMM provides a promising solution for the miniaturization of rotational modulation technology. In the future, we will further explore the integration of MEMS gyrocompass in fields such as indoor autonomous navigation and autonomous driving.

## Supplementary information

Structure and machining processes of MEMS traveling-wave micromotor

Supplementary Figure 1: The main structure of the MEMS traveling-wave micromotor

Supplementary Figure 2: The traveling wave

Supplementary Figure 3: The fully integrated MEMS processing flow of the micromotor

Supplementary Movie 1: Specific characterization of the driving performance of the traveling-wave micromotor

Supplementary Movie 2: The position control process of the multi-position braking control system

Supplementary Movie 3: The software of the upper computer and sampling process of the proposed gyrocompass
