# Supplementary material for: A MEMS traveling-wave micromotor-based miniature gyrocompass
Source: Microsyst Nanoeng. 2025 Feb 18;11:27. doi: 10.1038/s41378-025-00868-9 (PMC11836240; doi:10.1038/s41378-025-00868-9)
Supplement: Supplementary file 1 — Structure and machining processes of MEMS traveling-wave micromotor [file 41378_2025_868_MOESM1_ESM.pdf]

Structure and machining processes of MEMS traveling-wave micromotor[1-3]

The main structure of the used MEMS traveling-wave micromotor is stacked from top to bottom by a top-powered Pt electrode, a lead zirconate titanate (PZT) layer, a bottom-grounded Pt electrode, and the top silicon substrate, as shown in Supplementary Fig. 1. The outer and inner diameter of the micro-stator circular ring are 6,000 μm and 2,000 μm, respectively. The top silicon substrate has a thickness of 35 μm, providing support to the layers above it. The PZT layer, which is responsible for providing the vibration of the structure as in Eq. (1) through the inverse piezoelectric effect, has a thickness of 5 μm. Above and below the PZT layer, two thin Pt electrode layers are only 0.1 μm thick. These layers collectively contribute to the functionality of the structure. Both the PZT layer and the top silicon substrate have the same planar dimensions, which define the size of the micro-stator.

After the superposition of the two standing waves, a traveling wave is generated on the micro-stator, as in Eq. (2). It can be understood from the above formula that the direction of motion of the traveling wave can be changed by changing the phase difference of the two standing wave drive voltages, and the amplitude and frequency matching degree of the two standing wave modes is particularly important for the synthesis of high-quality traveling waves, and the quality of traveling wave is the core issue of motor drive because it determines the contact force and operating performance. Based on the traveling wave, the particle on the stator surface can move along the elliptical motion trajectory, as shown in Fig. 1g and Supplementary Fig. 2. The tangential velocity of the particle is shown in Supplementary Eq. (1), where *h* is the distance from the stator neutral surface to the stator surface:

$$v = -hA_z k\omega_n sin(k\theta \mp \omega_n t) \, . \quad (1)$$

When the stator serves as a driving unit, the rotor is fixed axially to the stator under the action of a vertical preload and is driven by friction caused by the elliptic movement of the stator surface. Based on the tangential velocity of the above formula, it can be seen that the maximum speed that the rotor can reach is $v_{max}=-hA_z k\omega_n$, and the direction of movement of the rotor is opposite to that of the traveling wave.

A fully integrated MEMS processes were applied to fabricate the traveling-wave micromotor. Mass-produced SOI wafers were used as the substrate where the thickness of the handle layer, buried SiO_2 and the top silicon substrate are 400 μm, 1 μm, and 35 μm respectively. The bottom-grounded Pt electrode was deposited on the substrate by sputtering. About 5 μm of PZT thin film was sputtered afterward, working as the piezoelectric actuating material. The top-powered Pt electrode was then deposited above using the same process as the bottom electrode. Sputtering Au coating on top of pt electrodes for gold wire connection between chip pads and external pads. Au During the structural patterning, different etching methods were employed. For Si and SiO_2, deep reactive ion etching (DRIE) was used. Ion beam etching was favored for metal etching, and wet etching was applied for PZT etching. According to Supplementary Fig. 3, the top electrode, PZT, bottom electrode, and top silicon substrate were patterned orderly to form the ring micro-stator. DRIE was conducted afterward for back etching and structural release before the suspended micro-stator membrane structure finally formed. The handle layer and buried SiO_2 were used as the supporting substrate structure of the micro-stator. The micromotor chip is formed by connecting the micro-stator with the supporting substrate through a plurality of supporting beams.

The processed MEMS traveling-wave micromotor chip can be referred to in Figs. 1d, 1e, and 3a. We have done a lot of tests on the driving performance of the traveling-wave micromotors, and the specific characterization of their driving performance can be found in Supplementary Movie 1. In

addition, the position control process of the multi-transposition braking control system can be seen in Supplementary Movies 2. Meanwhile, the software of the upper computer and the sampling process of the proposed gyrocompass can be found in Supplementary Movie 3.

Supplementary References

1. Xie, J. et al. Research on the Micro-Stator of a MEMS Traveling-Wave Motor Based on the Air-Damping Loss Model. *IEEE Sens. J.* **23**, 30202-30212 (2023).
2. Xie, J. et al. A Stator Design of Traveling-Wave Ultrasonic Micromotor. In *2022 IEEE 5th International Electrical and Energy Conference (CIEEC)* 197-201 (IEEE, 2022).
3. Qin, F. et al. A Novel PZT-Based Traveling-Wave Micromotor With High Performance and Unconstrained Coaxial Rotation. *J. Microelectromech. Syst*. **27**, 635-642 (2018).
