# Supplementary figures and images for: A MEMS traveling-wave micromotor-based miniature gyrocompass
Source: Microsyst Nanoeng. 2025 Feb 18;11:27. doi: 10.1038/s41378-025-00868-9 (PMC11836240; doi:10.1038/s41378-025-00868-9)

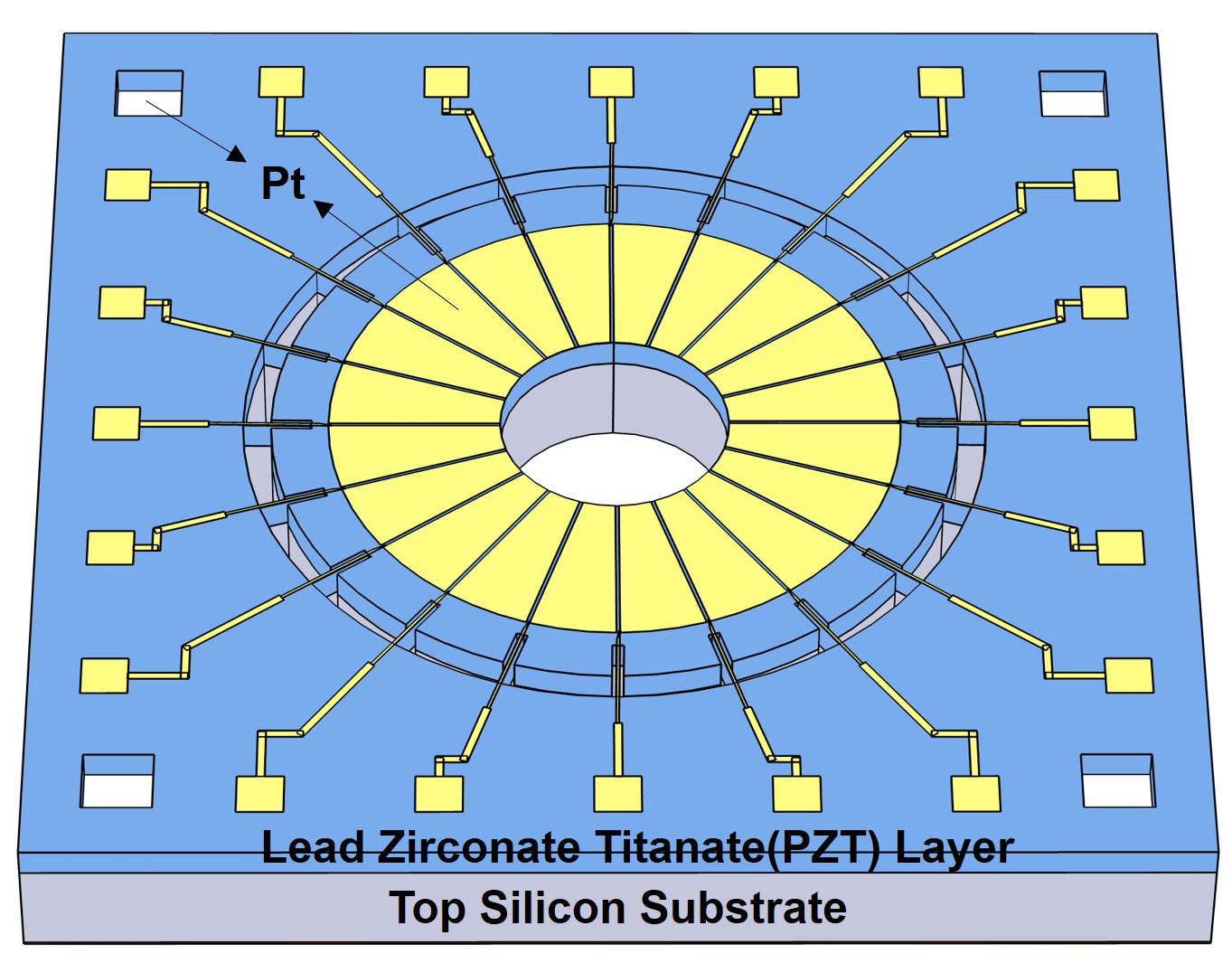

Supplement: Supplementary file 2 — Supplementary Figure 1: The main structure of the MEMS traveling-wave micromotor [file 41378_2025_868_MOESM2_ESM.jpg]

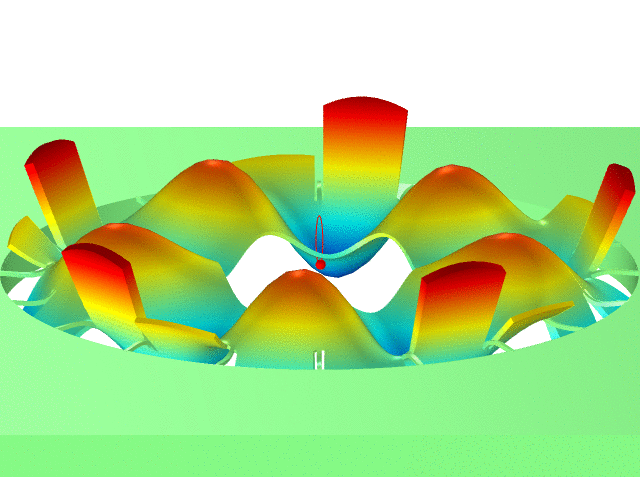

Supplement: Supplementary file 3 — Supplementary Figure 2: The traveling wave [file 41378_2025_868_MOESM3_ESM.gif]

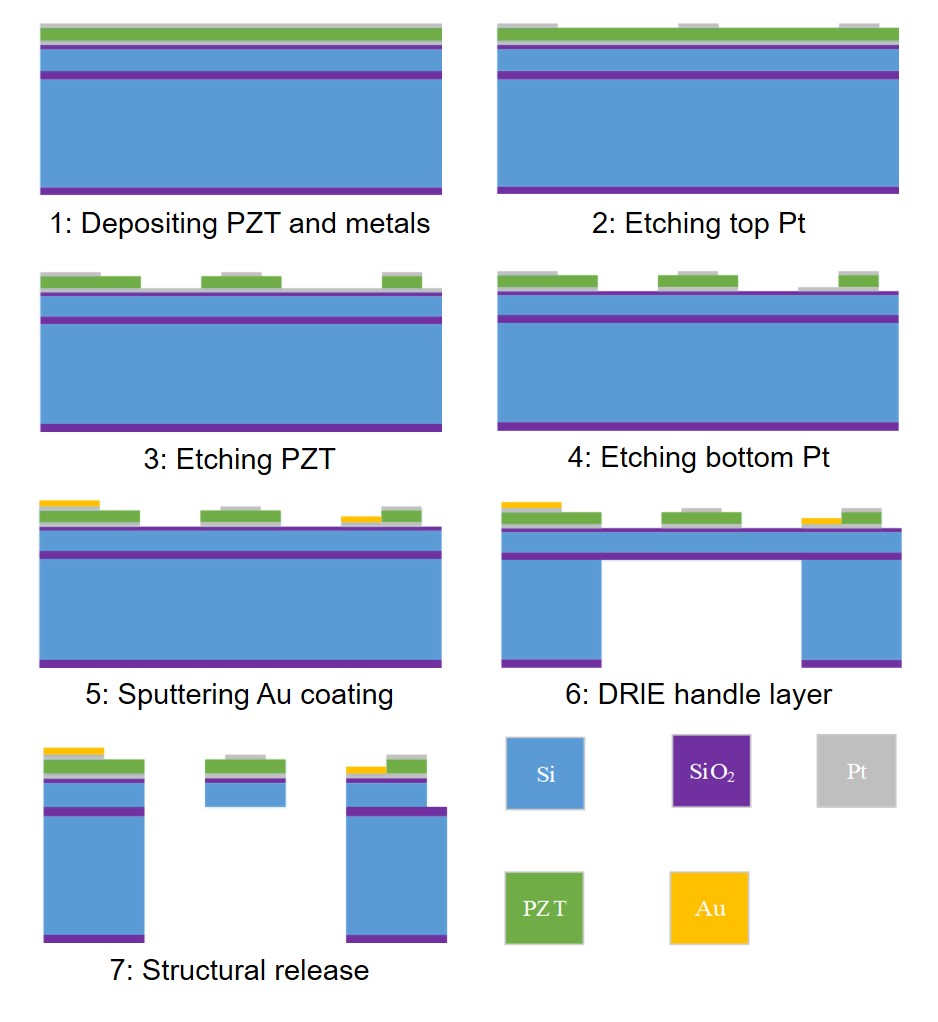

Supplement: Supplementary file 4 — Supplementary Figure 3: The fully integrated MEMS processing flow of the micromotor [file 41378_2025_868_MOESM4_ESM.jpg]
